# InTool Explorer: An Interactive Exploratory Analysis Tool for Versatile Visualizations of Neuroscientific Data

**DOI:** 10.3389/fnana.2019.00028

**Published:** 2019-03-11

**Authors:** Diana Furcila, Marcos García, Cosmin Toader, Juan Morales, Antonio LaTorre, Ángel Rodríguez, Luis Pastor, Javier DeFelipe, Lidia Alonso-Nanclares

**Affiliations:** ^1^Laboratorio Cajal de Circuitos Corticales (CTB), Universidad Politécnica de Madrid (UPM), Madrid, Spain; ^2^Centro de Investigación Biomédica en Red sobre Enfermedades Neurodegenerativas (CIBERNED), Madrid, Spain; ^3^Facultad de Psicología, Universidad Nacional de Educación a Distancia (UNED), Madrid, Spain; ^4^Escuela Técnica Superior de Ingeniería Informática, Universidad Rey Juan Carlos, Madrid, Spain; ^5^Center for Computational Simulation (CCS), Universidad Politécnica de Madrid (UPM), Madrid, Spain; ^6^Graphext, Madrid, Spain; ^7^Escuela Técnica Superior de Ingenieros Informáticos, Universidad Politécnica de Madrid (UPM), Madrid, Spain; ^8^Department of Functional and Systems Neurobiology, Instituto Cajal (CSIC), Madrid, Spain

**Keywords:** data visualization, dynamic analysis, interactive tool, multiscale, software

## Abstract

The bottleneck for progress in many research areas within neuroscience has shifted from the data acquisition to the data analysis stages. In the present article, we propose a method named InTool Explorer that we have developed to perform interactive exploratory data analysis, focusing on neuroanatomy as an example of its utility. This tool is freely-available software that has been designed to facilitate the study of complex neuroscience data. InTool Explorer requires no more than an internet connection and a web browser. The main contribution of this tool is to provide a user-designed canvas for data visualization and interaction, to perform specific exploratory tasks according to the user needs. Moreover, InTool Explorer permits visualization of the datasets in a very dynamic and versatile way using a linked-card approach. For this purpose, the tool allows the user to select among different predefined card types. Each card type offers an abstract data representation, a filtering tool or a set of statistical analysis methods. Additionally, InTool Explorer makes it possible linking raw images to the data. These images can be used by InTool Explorer to define new customized filtering cards. Another significant contribution of this tool is that it allows fast visualization of the data, error finding, and re-evaluation to establish new hypotheses or new lines of research. Thus, regarding its practical application in the laboratory, InTool Explorer provides a new opportunity to study and analyze neuroscience data prior to any statistical analysis being carried out.

## Introduction

The bottleneck for progress in many research areas within neuroscience has shifted from the data acquisition to the data analysis stages. The availability of more powerful microscopes and techniques to explore the brain has provided neuroscientists with a wealth of data that is difficult to fully analyze, due to both its volume and complexity (Kandel et al., 2013; DeFelipe, 2017).

Exploratory Data Analysis refers to a set of techniques originally developed to display data in such a way that those features that might be considered particularly relevant for a given analysis will become apparent. This utility provides the user with a different viewpoint, which could help either to decide the type of analysis to be performed, or to propose new hypotheses. The traditional methods require the hypothesis to be proposed before acquiring the data. However, Exploratory Data Analysis displays the data in a dynamic way which may give rise to new questions to be answered. Thus, the main goal of exploratory analysis techniques is helping users to better understand their data by identifying patterns, trends and outliers, in order to generate new hypotheses, formulate models and extract new data (Tukey, 1977). Widely used analysis tools such as R (Ihaka and Gentleman, 1996), SPSS (Arkkelin, 2014) and STATISTICA (TIBCO Software Inc., 2018) provide a low-level interface that hinders the use of such tools by non-expert users and prevents rapid specification of interactive data analysis. Data visualization and interaction are key components in Vega-Lite (Satyanarayan et al., 2016), D3 (Bostock et al., 2011) and Protovis (Bostock and Heer, 2009) declarative languages. In this context, declarative languages and visualization grammars provide powerful environments for engineers to design interactive visualization systems. However, these environments require programming skills, and are not meant to be used by final users, preventing their use in dynamic exploratory analysis workflow. Other applications, such as Tableau (based on Polaris Stolte et al., 2002) and Visflow (Yu and Silva, 2017) offer a much simpler interface, but they are closed platforms which are not specifically adapted to the needs of neuroscience. Besides, most of these tools do not support statistical analysis, which limits their range of applications. Therefore, it is becoming necessary to advance in the development of new methods and interactive exploratory analysis tools for neuroscientists to improve their data analysis results. In this work, we propose a tool to close the gap between flexibility and user-friendliness. A recent, detailed and exhaustive survey on visualization tools can be found in Mei et al. (2018).

Different fields of neuroscience require different approaches for visualization and data analysis. Here, we shall focus on neuroanatomy as a proof of concept. Within this discipline of neuroscience, numerous factors must be taken into account for data analysis. Such factors that contribute to making this task extremely challenging include:

– Age, species/cases, and conditions of the sample (healthy vs. ill, type and extent of disease, post-mortem interval, type of fixation, etc.).
– Brain region analyzed (neocortex, hippocampus, thalamus, cerebellum, etc.).
– Number and variety of structures/components to be studied in a particular experiment.
– Level of detail of the features analyzed: neuronal circuits, neurons, dendritic arbors, synaptic microcircuits, synapses, molecules, etc.
– Nature of the imaging technique (SPECT, PET, EEG/MEG, fMRI, optical microscopy, electron microscopy).
– Spatial scale or temporal resolution of the sampled data.

In general, the possible relationship between these different data sets is difficult to realize or could go unnoticed unless a particular hypothesis is proposed and then tested with the appropriate tools. For example, the effects of age, post-mortem intervals, and type of fixation may have an influence on the quantitative determination of the number of neurons in a particular region of the brain labeled with different techniques, such as Nissl staining or immunocytochemistry using antibodies against the neuronal nuclear antigen NeuN (e.g., Werner et al., 2000; Montero, 2003; Gonzalez-Riano et al., 2017). This article describes “InTool Explorer” (Interactive Tool Explorer), a Web-based exploratory analysis tool that is freely available software and has been designed to deal with the problems that neuroscientists commonly face when analyzing complex data. This tool was tailored to perform interactive exploratory analysis on generic tabular data. In order to tackle this issue, InTool Explorer was designed to allow a versatile configuration of interaction, visualization and exploration, also making it possible to make use of filters and different views of easily configurable cards. The following list sums up the main contributions of the tool presented here:

– **Versatile, dynamic and user guided configuration of the visualizations and interactions**. One of the major contributions of this work is to propose a new computational solution for improving the workflow in neuroscience that offers end users the possibility to dynamically adapt visualization, analysis and filtering operations to their data and current task.
– **Coordinated views**. In order to display data and interact with them, the linked-card approach gives users the opportunity to easily select among visual widgets (“cards”), which work in a coordinated manner (cards are co-dependent).
– **Extensibility**. InTool Explorer was designed in such a way that new features can be easily included. Considering the fast development of the acquisition techniques, it is very difficult to predict the future needs of the field. In this sense, the technologies and the software architecture used were carefully designed to allow the integration of new cards, which will work in synchronization with the previously defined cards.
– **Visual interaction**. InTool Explorer allows the inclusion of raw images—such as MRI, SPECT and/or histological microphotographs—in the interactive exploratory analysis task. Commonly, data can be extracted from images or, the tabular data may have images attached. Thus, InTool Explorer gives the user the opportunity to add these images to the data analysis process and to use them for interaction with the data.
– **Collaborative and portable**. Standalone software tools are currently the ones most commonly installed in neuroscience laboratories. InTool Explorer has been designed to satisfy standalone users, as well as local storage of their data. Moreover, software architecture is flexible enough and fully portable to allow a web-based multiuser installation where data can be stored in remote servers, which are accessible from any system, requiring no more than a web-browser and an internet connection.

## Materials and Methods

The input data for testing the usefulness of InTool Explorer consist of the data analysis of clinical-pathological information obtained from Alzheimer’s disease (AD) patients.

Histological data from AD patients were obtained *via* the detailed histological analysis of labeled neurons (normal and pathological) and amyloid-β (Aβ) plaques, in different areas of the hippocampal formation, from a cohort of AD patients previously examined (Furcila et al., 2018). Clinical data were collected from the same hospitals and medical centers that provided the AD patient brain tissue. These samples were obtained from two sources: Banc de Teixits Neurològics from Hospital Universitari Clinic de Barcelona (Spain) and Banco de Tejidos Fundación CIEN (Madrid, Spain), following the guidelines of the Helsinki Declaration and with the approval of the local Ethical Committees.

## Description of Intool Explorer

### Overall Design

The tool was drafted with the aim of providing users with tools that facilitate the understanding of their data. In this regard, the paradigm of visual data exploration (Shneiderman, 1996) proved useful: “overview first, zoom and filter, then details on demand.” Nevertheless, facilitating the visualization data does not mean putting aside traditional procedures that could improve the analysis. InTool Explorer has been created using a User-Centered Design (UCD) methodology, since it is the approach that best suits the problem of applying visualization methods to a set of users from a specific domain which has not been exposed to this kind of technique. The implementation was performed using an agile development method similar to *scrum* because the principles proposed in this method (Rising and Janoff, 2000) are in line with the UCD philosophy. The final proposed design was based on incremental prototyping. This approach reinforces the user role in the design, implementation and testing of the system usability in each iteration.

The proximity and accessibility to final users fitted very well with the methodology; getting them more involved in the design process was an additional advantage. Moreover, the characterization and problem abstraction stages were performed using a mixture of methods including interviews and observations, although contextual inquiries proved to be the most effective approach.

### Software Distribution Details

Currently, two versions of InTool Explorer are available at: http://cajalbbp.es/intoolexplorer_web. Page links to the online web-based version and to the Windows standalone version can be found on this website.

The online version does not require a download or local installation to start working with the tool. The system has been fully tested with the most popular browsers, namely, Google Chrome, Microsoft Edge and Mozilla Firefox. InTool Explorer requires a very simple registration procedure where users provide a login identifier and email. This registration process allows neuroscientists to have access to their datasets from any PC *via* an internet connection and makes sharing their data possible. In addition, it allows ciphering of the data and the communications to guarantee data protection and comply with privacy laws.

Briefly, the application is designed to work with generic tabular data (cvs and xls) and standard 2D image formats (png, jpeg, tif, bmp, etc.). Tabular data have to be arranged in one data sheet. The elements of the dataset must be sorted into rows and ideally each column must contain all the values of a given variable, but the system is robust enough to deal with incomplete data.

After the login process, the first step is to load data using the File menu. If users have already uploaded data, they can open them from the server directly (“Open from server”), or upload local data to the server using the order: “Import local files to server.” The user can also append a new set to the current one (“Add to current”), and save the current dataset in the server (“Save on server”). In addition, variables of the dataset are automatically categorized, which is a very helpful utility to save time in operations that are necessary for the correct analysis of the data, but that are generally beyond the scope of the study. Moreover, the user can manually modify this categorization and remove, shuffle and rename variables editing the dataset schema (option Edit Schema). This also makes it possible to merge the dataset coming from different sources during a working session.

Finally, the Analysis widget allows users to locally save the state of the current exploratory analysis or load a previous saved state (active cards, filters, etc.). This allows the user to start a working session and finish it later working either on the same computer or on a different one.

### Linked-Card Views

**Figure 1:**
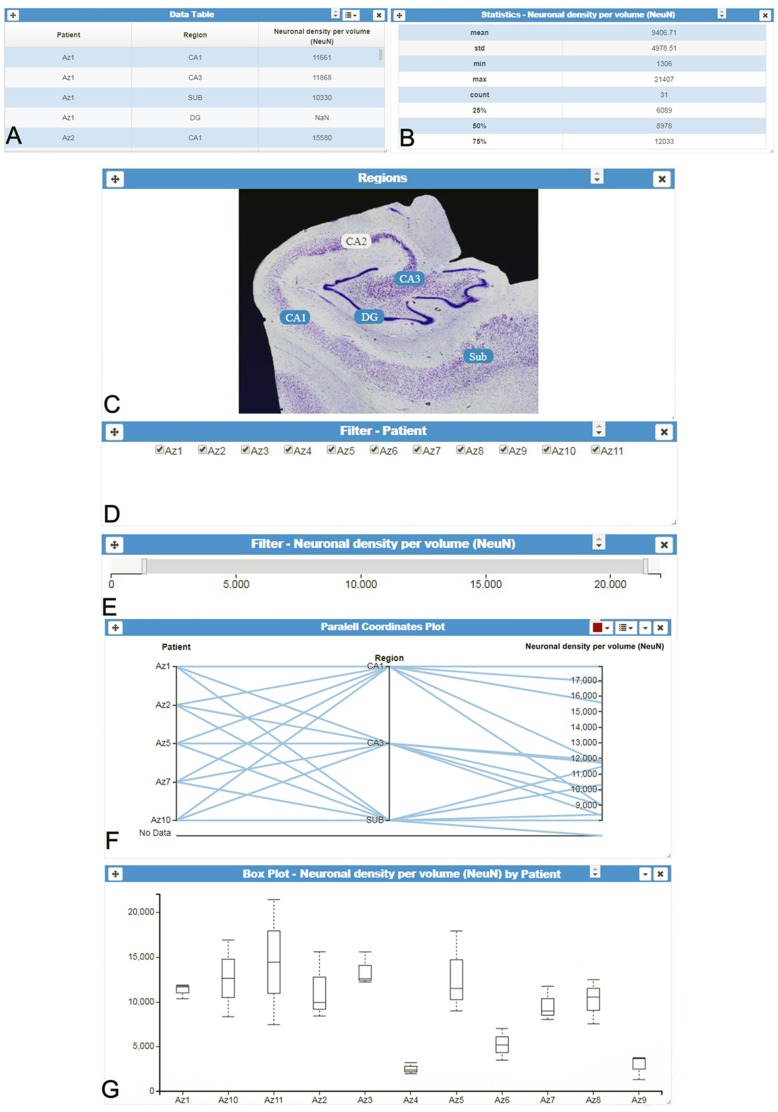
InTool Explorer interface. **(A)** Tabular data and **(B)** Column statistics card of the variable “Neuronal density per volume (NeuN).” **(C–E)** Different filter cards for spatial, categorical and quantitative variables: “Regions,” “Patients” and “Neuronal density per volume (NeuN),” respectively. **(F)** Interactive parallel coordinates plot card showing some selected variables [“Patients,” “Region” and “Neuronal density per volume (NeuN)”]. **(G)** Box plot card of selected data [“Neuronal density per volume (NeuN)” per “Patient”]. All visualized spatial objects are dynamically selected by users and updated according to user’s interaction with filters (linked-cards). “DG, dentate gyrus; CA1–CA3, hippocampal fields; SUB, subiculum.”

One of InTool Explorer’s main contributions is to customize visualizations, interactions and statistical analysis, and to adapt them to the particularities of the data. In order to achieve this goal, the tool employs a coordinated view system based on linked-card views. The linked-card views are the main functional units of the system, allowing interaction, visualization and analysis operations. Users can select from different predefined cards and arrange them freely in the main window (or interface) of the application. Cards can be rearranged on the canvas and resized at any time as required by users. All the selected cards work in a coordinated manner; operations performed in a given card propagate automatically along the other cards shown in the main window layout. Figure 1 shows an example of the user interface with several linked-cards. The linked cards can be classified into four different groups:

1. **Filtering cards**: InTool Explorer classifies the tabular dataset variables into three groups: categorical, ordinal and quantitative. The users can select any of these variables to perform searching and filtering operations. Each new filter is presented in a new card with interactive controls adapted to the nature of the variable. Categorical values are selected with checked-boxes, and quantitative and ordinal values with dual knob range sliders.
2. **Visualization cards**: users can select a suitable view based both on the task to perform, and on the data to manage. Interaction with card filters is linked to all visualization cards, and figures are updated when users change filter parameters. In addition, some of the visualization tools provide selection and/or filtering capabilities that are linked to the active cards shown in the main window. Six popular views have been selected to cover common tasks: parallel coordinates plot (Inselberg and Dimsdale, 1990), parallel set (Kosara et al., 2006), radar chart (also known as radial bar chart, spider chart, polar chart, web chart, or star plot; Chambers et al., 1983), scatter plot (Friendly and Denis, 2005), box plot (Tukey, 1977) and raw tabular data.
3. **Domain-specific data cards**: accessing original raw data is often required by users (original domain; e.g., Figures 1A,C) while exploring the processed data (transformed domain; e.g., Figures 1E–G). For example, in some situations, sample preparation and the analysis process introduce errors or noise in the transformed domain. These problems may be translated into different patterns in the visualization cards. The original data cards allow the user to check the original data at any point during the analysis. InTool Explorer provides a mechanism to associate this data to the tabular data. In addition, these cards can be used to create new spatial interaction cards (see Section “Visualization Cards” for further detail).
4. **Statistics cards**: as mentioned above, InTool Explorer was mainly designed to perform exploratory visual analysis in order to generate new hypotheses and models, as well as to drive the extraction of new data. In this regard, InTool Explorer is focused on providing an extensible set of visualizations. With the aim of enhancing the visualization capabilities with statistical analysis functionalities, we have integrated our system with R. Our server runs scripts in R in order to support statistical cards, such as Correlation test and Comparison of means.

It should be noted that the system architecture was designed to include additional visualization cards relatively easy (see section “Analysis of Quantitative Variables”).

### Visualization Cards

**Figure 2:**
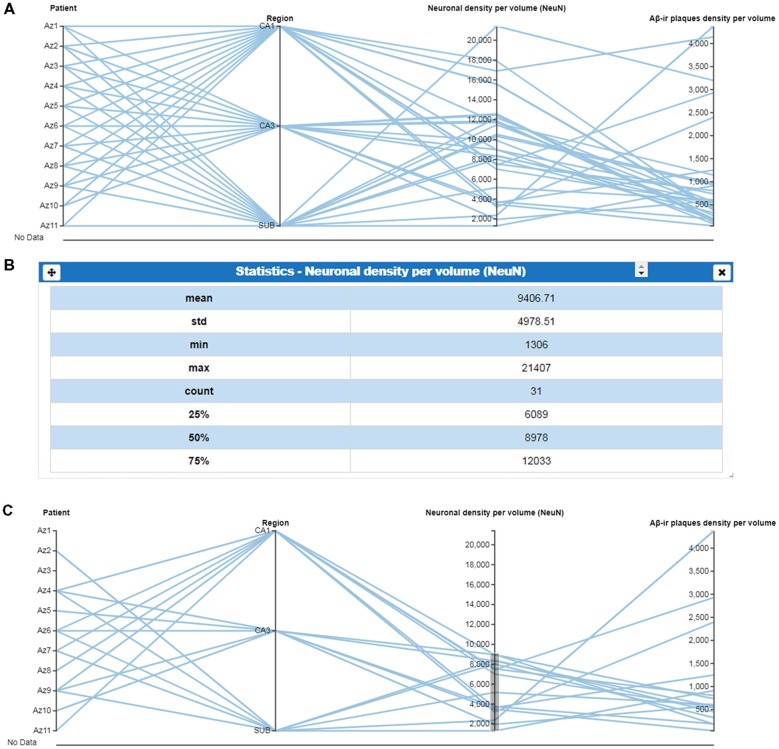
Use of parallel coordinates plots. **(A)** Parallel coordinates plot of selected variables, in columns. **(B)** Column statistics of the variable “Neuronal density per volume (NeuN)” which facilitates the selection of specific values. **(C)** Parallel coordinates plot using ranged values for “Neuronal density per volume (NeuN)” to visualize the regions or patients corresponding to a selected range (mean values showed in **B**). This selection facilitates the visualization of a possible relationship with the variable “amyloid-β (Aβ)_-ir_ plaques density per volume.” CA1–CA3, hippocampal fields; SUB, subiculum.

As previously mentioned, most visualization cards allow interactive operations with the data shown. Users may modify the current view using interactive controls. For example, the parallel coordinates plot is ideal for representing and comparing multidimensional data. Users can define, on each parallel axis, a filter implemented with dual knob range sliders. By defining simultaneous filters over several parallel axes, users can combine several selection criteria, using the parallel coordinates card. This functionality replaces the need to add new filter cards for each variable and partly alleviates the issue of parallel coordinates not scaling as well as other visual representations (Nguyen and Rosen, 2017). In the same way, the performance of the parallel coordinates plot, while studying the correlation between variables, has been improved by allowing the user to reorder (shuffle) the parallel axis’ coordinates. Currently, users can also include new axes or remove them using a tab that can be displayed listing all the variables from the study. Moreover, when including a new variable in the plot, the variables already shown are highlighted in a different color. Finally, this card highlights data elements or groups at the request of the user. Figure 2 shows a parallel coordinates plot card, for which data below the mean (given by Statistics card in Figure 2B) were selected, and a range filter was defined over the quantitative variable “Neuronal density per volume (NeuN)” (Figure 2C).

**Figure 3:**
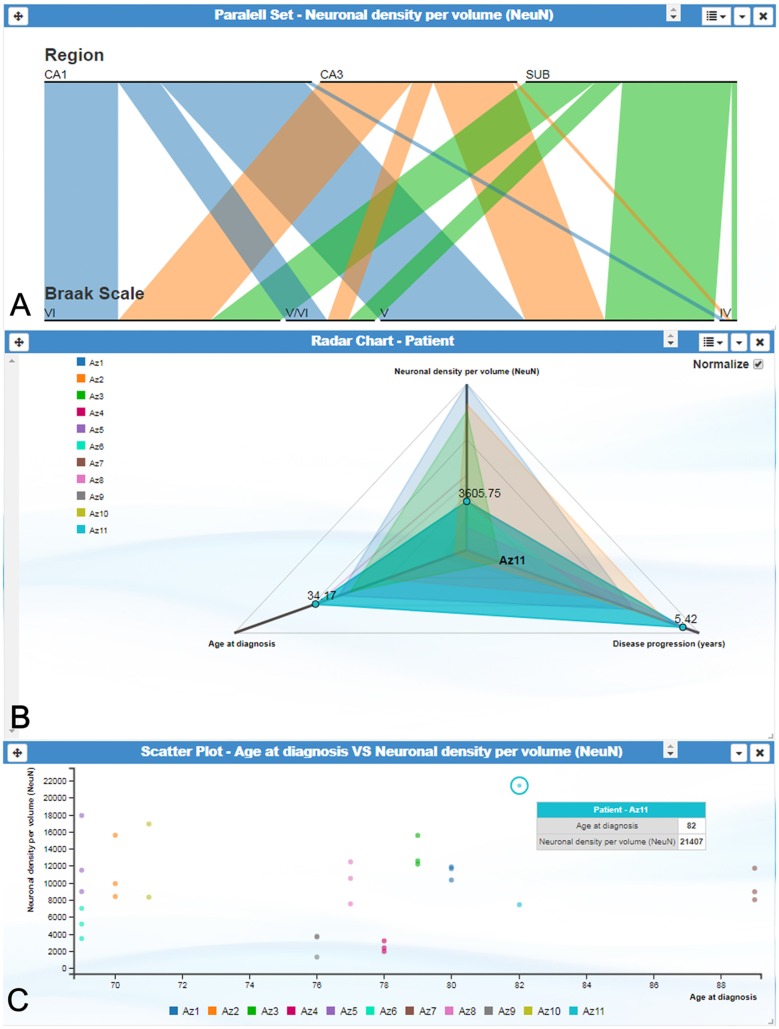
InTool Explorer interface showing several visualization cards.** (A)** Parallel set plot showing the variables “Region,” “Braak Scale” and “Neuronal density per volume (NeuN).” Each horizontal bar represents possible categories associated to each variable, and bar width indicates the proportional fraction of the total category. **(B)** Radar chart illustrating the relation between three variables: “Age at diagnosis,” “Neuronal density per volume (NeuN)” and “Disease progression (years).” All patients are visualized on the left side of the plot. This visualization card allows multiple comparisons in order to visualize similar or extreme values. Note patient Az11 (highlighted in blue), expressing distinctive values. **(C)** Scatter plot showing the relationship between the variables “Age at diagnosis” and “Neuronal density per volume (NeuN)” per patient. Note the extreme value highlighted with tooltip balloon (blue). This identification offers additional information about the selected value. Braak Stages (Braak and Braak, 1991): I-II (neurofibrillary tangles in entorhinal cortex and closely related areas); III-IV (neurofibrillary tangles abundant in amygdala and hippocampus and extending slightly into the association cortex); V-VI (neurofibrillary tangles widely distributed throughout the neocortex and ultimately involving primary motor and sensory areas). CA1–CA3, hippocampal fields; SUB, subiculum.

Other representations also allow user interaction. For example, parallel set plots can automatically rearrange the data shown when users shuffle each category according to their criteria (Figure 3). Similarly, data tables, radar charts and parallel sets can add and remove axes interactively at the request of the user. Scatter plots allow particular data elements to be selected and highlighted. When these interactions involve shared variables among different cards, the local changes performed on one card are propagated through the other cards. Additionally, scatter plots, radar charts and parallel sets display tooltip balloons with additional information on the selected variable.

### Domain-Specific Data Cards

**Figure 4:**
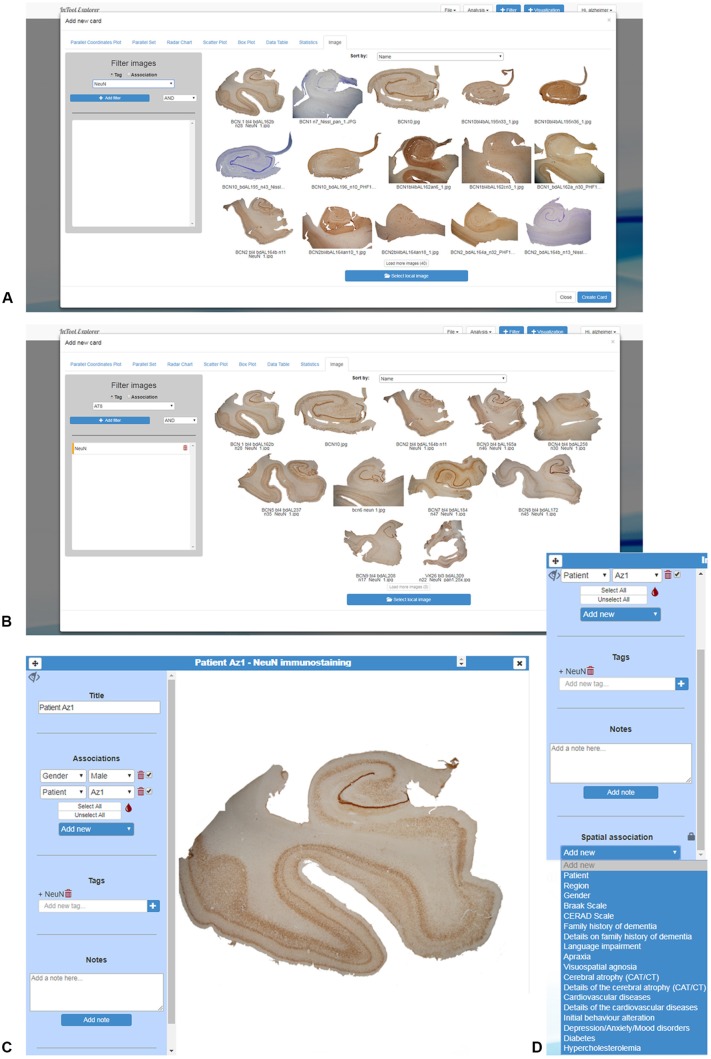
Domain-specific cards created in InTool Explorer from histological images of human brain samples from Alzheimer’s disease patients. **(A)** Uploaded images can be visualized all together. **(B)** As images can be filtered by tags, all images displayed correspond to the selected marker “NeuN.” **(C)** The created card shows information regarding the selected image: title, variables associated to the image, tags and notes, as well as “spatial association” (details in **D**). **(D)** This feature provides the facility to add visual filters of categorical variables to the selected image (see Figure 5 for more details).

As previously explained, the original data cards provide mechanisms to associate the original input to the data in the transformed domain. The other cards provide several tools to visualize tabular data. Tabular data is the most common type of data in the transformed domain, but it is rarely available in the original domain. Besides, in neuroscience, images are frequently the input data from which the tabular data is computed. In order to support data in the original domain, InTool Explorer allows 2D images to be included within the dataset. These images can be associated to the available tabular data and they can be connected to a particular value of an ordinal or a categorical variable (e.g., one image can be associated to a particular patient). In addition, images can be labeled using arbitrary tags defined by the user. Using an ordinal or categorical value and/or a tag, an image can be retrieved from the database (Figure 4).

**Figure 5:**
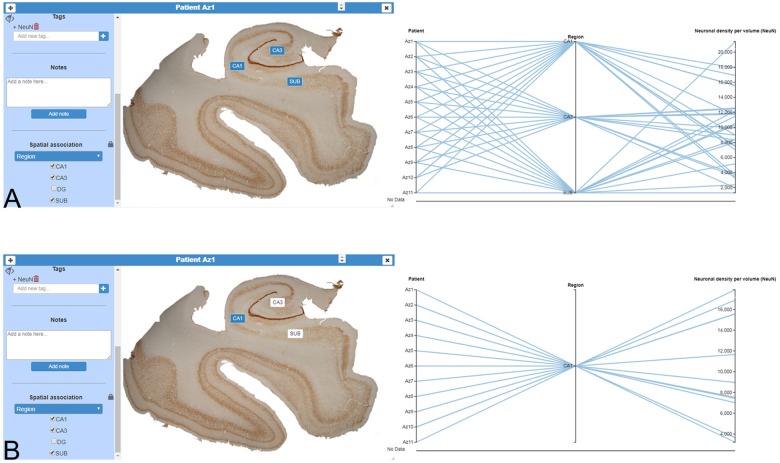
Domain-specific cards for spatial filtering. **(A)** Image card of a hippocampal section immunostained for anti-NeuN from an AD patient (Patient Az1). The variable “Region” was added to the image card with the function “Spatial association,” as a filter for any kind of simultaneous visualization. On the right, a parallel coordinates plot was created to show the dynamic of the user interaction. **(B)** In the same image card displayed in **(A)**, a particular region has been selected (CA1, highlighted in blue). On the right, only filtered data appear in the parallel coordinates plot. CA1–CA3, hippocampal fields; SUB, subiculum.

Image cards can be used for filtering and selecting. In the simplest interaction mode, scientists can use the values of the variable associated to an image to select or filter data. InTool Explorer is a sophisticated tool that allows neuroscientists to develop their own spatial filtering tools. Frequently, general visualization techniques for tabular data fail to represent the spatial structure of the data. In this work, we propose using the original input images to create user defined spatial filter cards. The original data card can be used to add categorical data values to specific parts of the image, and the user can then click on those areas to perform filtering operations. This spatial filtering tool allows InTool Explorer to be adapted to a specific dataset (Figure 5).

### Analysis of Quantitative Variables

**Figure 6:**
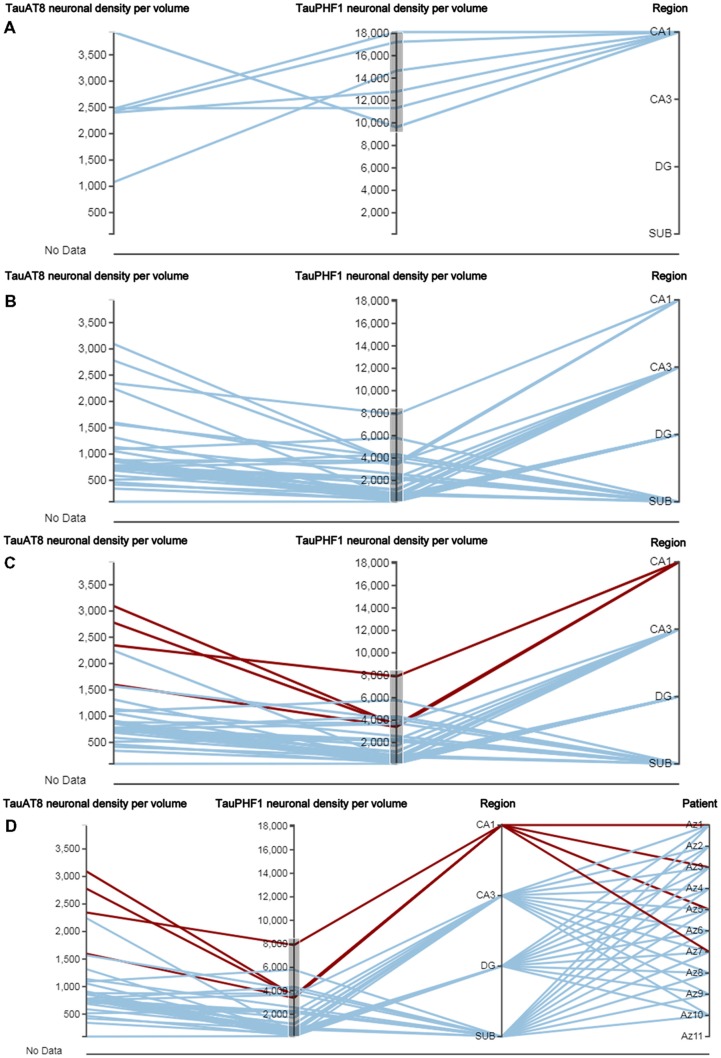
Parallel coordinates plots from AD patients. This type of plot has some particular characteristics, such as the selection of a certain range of data in the axis of any quantitative variable **(A,B)**, as well as the highlighting of concrete relationships between variables (**C,D**; see CA1 region selected) and the possibility of adding new variables in the same visualization card (“Patient” was added in **D**). DG, dentate gyrus; CA1–CA3, hippocampal fields; SUB, subiculum.

**Figure 7:**
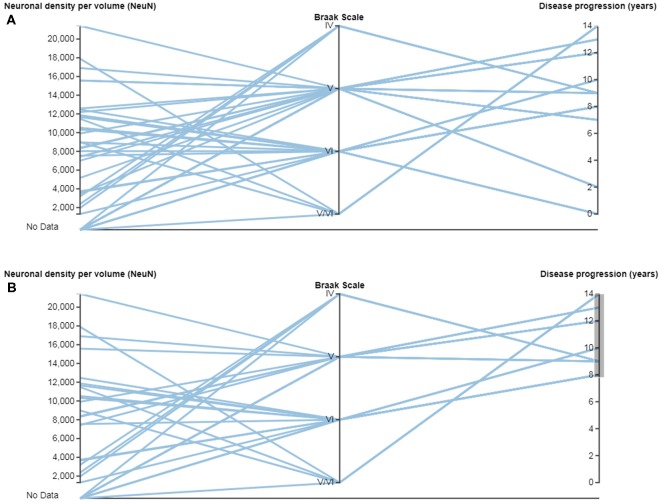
Parallel coordinates plots with quantitative and categorical variables from AD patients. **(A)** Relations can be made between quantitative variables such as “Neuronal density per volume (NeuN)” and “Disease progression (years),” and categorical variables such as “Braak Scale.” **(B)** Note that a range of data can be selected in the variable “Disease progression (years),” to examine possible relationships. Braak Scale (Braak and Braak, 1991): I-II (neurofibrillary tangles in entorhinal cortex and closely related areas); III-IV (neurofibrillary tangles abundant in amygdala and hippocampus and extending slightly into the association cortex); V-VI (neurofibrillary tangles widely distributed throughout the neocortex and ultimately involving primary motor and sensory areas).

In neuroanatomy, the quantitative analysis of variables is particularly relevant; for example, InTool enables the analysis of quantitative variables such as “Neuronal density per volume (NeuN)” which shows huge inter-individual variability. Regarding this variable, the filter function in a parallel coordinates plot can be applied and variable values can be associated to each patient and to certain hippocampal regions (Figure 6), which facilitates visualization and exploration. In addition, a value selection of any quantitative variable can be carried out (Figure 6C), to examine a particular range of data, which can be denoted in a different color. Moreover, with InTool Explorer, it is possible to add any variable to a given plot enhancing the interaction of the user. For example, if a user wishes to investigate patients showing a particular value, the variable “Patient” can be added to the parallel coordinates plot (Figure 6D). Particular patients and their particular characteristics can then be further explored to examine inter-individual variability as well as possible relationships with the histopathological characteristics. Thus, InTool Explorer provides a quick visualization of atypical cases within a dataset. Regarding qualitative variables, like clinical-pathological data of patients, InTool Explorer may combine any of them with quantitative data, producing an integrated analysis. To illustrate this utility, the relation between the histopathological variable—“Neuronal density per volume (NeuN)”—and the clinical features—“Disease progression” and “Braak stage”—was explored (Figure 7).

In summary, data from AD patients are very complex and diverse, and multidimensional visualization greatly facilitates the integration of any type of data as well as exploration of individual values within each variable. As has been shown, InTool Explorer allows users to dynamically configure visualizations and interactions in a very flexible and personalized way, selecting the techniques that best fit their data. The linked-card based approach lends support to this goal, by adjusting the layout of the filters and views according to the particular requirements of the task at hand.

### Statistics Cards

As previously mentioned, InTool Explorer was designed to simplify the visual exploratory analysis task of neuroscientists. Furthermore, the tool was intended to be extensible to face forthcoming challenges. Moreover, the tool needed to be flexible enough to solve the peculiarities of the different problems in this specific research area.

In this first version, we have implemented the most common statistical tests performed during a preliminary exploratory analysis.

**Figure 8:**
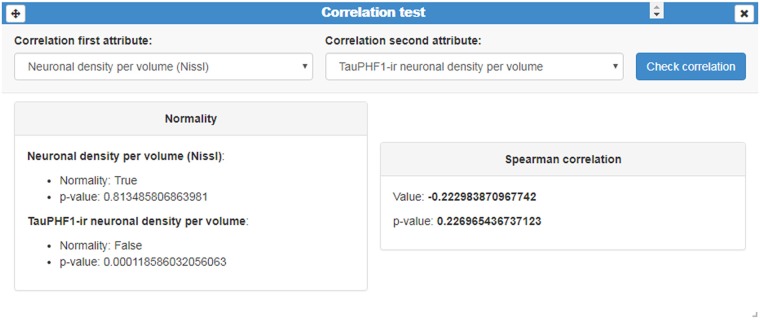
Correlation test card. This card displays an example of a correlation analysis of the quantitative variables “Neuronal density per volume (Nissl)” and “TauPHF1 neuronal density per volume.” As normality was not met, InTool Explorer estimates the Spearman’s rank correlation coefficient and its associated *p*-value.

**Figure 9:**
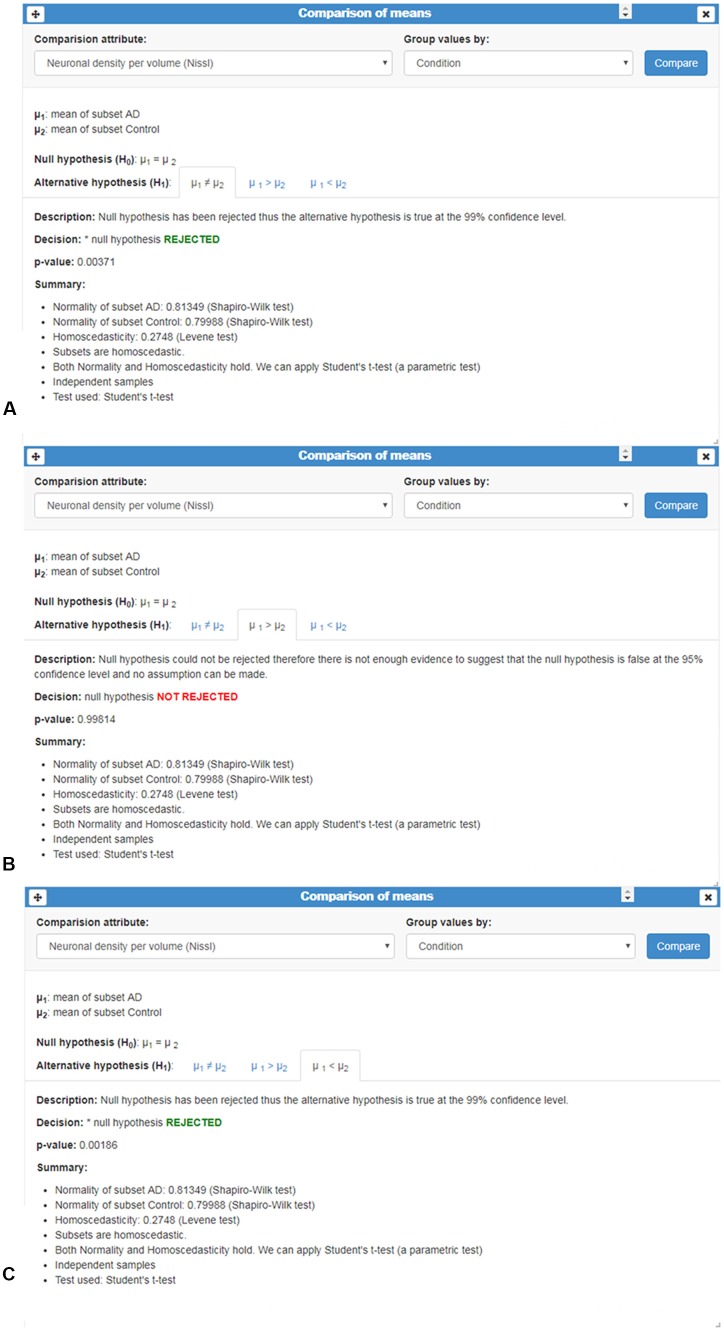
Comparison of means card. This card shows an example of the comparison of the qualitative variable “Neuronal density per volume (NeuN)” grouped by the categorical variable “Condition.” Number of cases, normality and homoscedasticity were evaluated to perform an appropriated test; in this example, Student’s *t*-test was applied. **(A–C)** Analysis is shown in three different tabs corresponding to the results of comparing whether: **(A)** the means are equal (μ1≠μ2); **(B)** one sample is less than the other μ1>μ2; and **(C)** one sample is greater than the other (μ1<μ2). In this example, *p*-values revealed significant mean differences: means were not equal **(A)** and, in particular, the mean of AD group was smaller than the mean of the Control group **(C)**.

These tests were implemented in three card types: (i) a column statistics card; (ii) a correlation test card; and (iii) a comparison of means card:

(i) *Column statistics*: this card provides a brief description of data distribution, providing the mean, standard deviation, size (n), as well as minimum and maximum values (Figure 2B). No previous statistical knowledge is needed to use any of the cards. All of them automatically select the test to be performed, taking into account the nature and size of the analyzed sample. The cards provide a detailed explanation of the process followed, allowing users to communicate their results.
(ii) *Correlation test*: this card performs a correlation analysis of quantitative random variables. First, we check data normality, using a Kolmogorov-Smirnov Goodness-of-Fit test. If normality holds, two tests can be computed: Pearson’s correlation coefficient and Spearman’s rank correlation coefficient test. If not, we only calculate the Spearman’s rank correlation coefficient (Figure 8) Pearson’s coefficient offers a measure of linear correlation between two variables, while Spearman’s rank coefficient assesses if two variables can be described by monotonic function (linear or non-linear).
(iii) *Comparison of means*: this card compares two or more unpaired variable distributions (Figure 9). Depending on the number of variables and whether normality and homoscedasticity holds, several tests can be performed: Student’s *t*-test, Welch’s *t*-test, Kolmogorov-Smirnov, One-way ANOVA or Kruskal Wallis. In addition, to test prove the utility of this card with a larger data set, as a proof of concept, we selected data from 8,900 human dendritic spines (Benavides-Piccione et al., 2012). In the present study, we compared the dendritic spine length between the apical and basal dendrites obtaining significant differences, in agreement with the findings of Benavides-Piccione et al. (2012).

Finally, in order to guarantee the extensibility of this module, InTool Explorer is fully integrated with R to support statistical analysis cards.

### System Architecture and Technologies Used

In order to make the tool portable and to facilitate cooperation among different neuroscientists, a multi-user Web platform was implemented. Furthermore, since InTool Explorer will be released with a policy of it being freely available and given that it is expected to be widely used within the neuroscience community, a set of robust and widely tested free tools were selected.

The tool follows a client-server model and is divided into two layers: a backend and a frontend. Each of the components of these two layers was developed using the appropriate technology according to its required features. The frontend and backend exchange information through WebSockets (Wang et al., 2012).

The backend is in charge of accessing and processing the data and is made up of three modules (the Main Server, the Statistical Server and the Database). To improve the system portability, these three components run in a Docker virtual container (Docker Inc., 2018). Docker has implementations for MacOS, Linux and Windows making it possible to deploy the server in any of these systems. Furthermore, the use of this virtual container enabled the development of a standalone version of the system, developed for those users unwilling to upload their data to a remote server. With this goal in mind, a portable application called WeCo (Web Container) was implemented for Linux, Mac OS and Windows.

The Main Server is one of the centerpieces of the system. It receives and processes the frontend requests. Among other tasks, it is responsible for the synchronization of the other components; data management operations; filtering operations; and user authentication. It is implemented using a Python Simple Http Server and uses MongoDB for data manipulation (MongoDB Inc., 2018) and ZeroMQ sockets to communicate with the Statistical Server. InTool Explorer is designed to be used in the visual exploration of the data, prior to the statistical analysis. This component provides support to the basic Statistical Analysis cards included in the frontend. Although currently the tool offers limited capabilities, this module was implemented using R to increase the application functionality in the near future. It should be noted that R is a powerful environment for statistical computing that offers a wide variety of statistical techniques.

The frontend provides different visual data representations and data interaction tools. It was developed in a single module and it implements the linked-card model proposed in this article. As previously mentioned, each card contains a data representation and/or filtering tool. User interactions in one card are designed to affect other cards to address the need for all the data visualizations to be coordinated. In order to accomplish this requirement and to allow the functionality of the available cards to be extended, a publish/subscribe pattern was followed to send and receive events. When the user performs an action, the card broadcasts an event of a given type. The cards that should react to a given event must explicitly subscribe to this event. The senders do not know which cards will receives the event and the receivers do not need to know which card triggers the event. Motivated by the need to also ensure that the system is suitable for a wide range of potential future applications, the visual representations were rendered using D3 (Bostock et al., 2011), a powerful library of interactive visualizations. React-Bootstrap (MIT, 2018) is a library of frontend components that was used to allow the users to configure the visualization layout easily.

## Discussion

The complexity of the brain, together with the shortcomings of the instruments and techniques available to date, still hampers progress of the specific research carried out in the field of neuroscience (Kandel et al., 2013; Martone and Ascoli, 2013). Nevertheless, despite these technical difficulties, research in neuroscience is acquiring new tools for study and analysis, as well as improving those that already existed, with the aim of unraveling the complexities of brain organization (DeFelipe, 2010, 2017; Hagmann et al., 2010).

Visualization has proved to be a powerful tool in exploratory analysis (Tukey, 1977). Interactive visualization methods help users to reach unexpected conclusions beyond the insight provided by standard statistics software. In this regard, the declarative languages mentioned above, such as D3 (Bostock et al., 2011), Polaris (Stolte et al., 2002), Vega (Satyanarayan et al., 2016), Protovis (Bostock and Heer, 2009) and ggplot2 (Wickham, 2009) are becoming popular environments to develop customized exploratory data applications. These languages offer a flexible methodology for rapid prototyping, which is essential for investigating new approaches in the context of exploratory analysis. Unfortunately, these environments require coding skills and prevent the final user from defining their own visualization workflow.

Currently, there are high-level visual analysis tools that allow the users to customize their visualizations. One very well-known system is “Tableau,” which provides a graphical user interface to Polaris (Stolte et al., 2002), and supports statistical analysis with R. However, writing scripts in R is beyond the capabilities of most final users. “Autodiscovery” is another exploratory analysis tool that performs a predefined set of statistical tests to find correlations (Butler Scientifics SL, Barcelona, Spain). The results of these tests are then shown using several visualization techniques. This system allows very little interaction and the users have no control over the data analysis process. Dataflow architectures are common in scientific visualization and in data mining (Abram and Treinish, 1995). In general, they are closed platforms that cannot be extended to include new functionalities or specific customized options.

There are already some exploratory data analysis tools available for studying genomics or proteomics (Gentleman et al., 2004; Zeeberg et al., 2005; Lawrence et al., 2006; Buske et al., 2011), or even for exploring the brain anatomy at different levels of detail (for example, displaying fiber tracts or dendrograms Jianu et al., 2009 or brain atlases Sunkin et al., 2013). Similar techniques are being applied for the exploratory analysis of neuronal circuit simulation data (Nowke et al., 2013). In addition, Angulo et al. (2016) have developed BRAVIZ, which is software for exploratory analysis implemented for MRI datasets as well as transcranial magnetic stimulation (TMS) and clinical data. Although BRAVIZ is a very useful tool, it does not support a web interface which allows multiple experts from different laboratories to share data in the way that InTool Explorer does. In the context of neuroscience, Yeatman et al. (2018) described a web-based visualization tool for diffusion MRI data. Although this tool supports simple data analysis, it was not designed for data exploration but rather for story-telling (to understand and reproduce published findings). All of the previously mentioned tools lack the flexibility that InTool Explorer offers. One of the main advantages of our proposed tool is its capacity to dynamically adapt to specific requirements of the task and/or the dataset being studied. Moreover, these tools do not allow the creation of new spatial filters while the tool is being used—again, a task that is possible with InTool Explorer.

InTool Explorer provides interaction techniques with different data representations, which make use of the power of combining these simultaneous representations to facilitate the task at hand. InTool Explorer has been shown to improve the study of complex data obtained from the analysis of multifactorial neurological conditions, such as AD, the research of which generates a huge volume of data which are both difficult to analyze with simple statistical tools and challenging to interpret. Since data from multidisciplinary sources can be visualized altogether with InTool Explorer, a more detailed analysis can be achieved, opening up the possibility of a more accurate definition of the neurological disease.

From the software point of view, InTool Explorer’s architecture allows new functionalities to be integrated very easily. The current filter and visualization linked-cards are just an example of those that could be implemented. As mentioned above, all the possibilities offered by R packages are available, because this module is already integrated in our tool. However, we have focused our efforts on providing more flexibility to set up linked filters and visualizations in the interactive exploratory analysis stage instead of developing more automatized statistical computations. As has been described above, users are able to create new linked-cards such as the example used in this case study—the Regions filter. In this way, the spatial data present in raw images (e.g., of brain regions) can be linked to tabular data (such as the clinical dataset), with no additional demands in terms of the level of user expertise. No software dependencies or specific hardware requirements are needed to work with InTool Explorer. Only a web browser is required, and the user’s work can be shared with any laboratory around the world *via* the internet.

Regarding task abstraction, InTool Explorer covers all the tasks proposed by Munzner (2014)—except *Enjoy—*although we hope users will implicitly gain enjoyment from performing this task. Below are several examples that cover all of the categories of this taxonomy:

- Analyze:
  – Consume:
    * Discover: new knowledge has been gained from the use of InTool Explorer.
    * Present: figures extracted from the tool have been used to show and explain new insights to neuroscientists in several forums.
  – Produce:
    * Annotate: for example, additional user-specific images can be included as new spatial filters.
    * Record: screenshots or new subsets of data can be recorded from a working session.
    * Derive: statistics can be easily extracted from populations.
- Search:
  – Lookup: sometimes users know what they are looking for and where it is.
  – Locate: sometimes they know what they are looking for without knowing where it is.
  – Browse: users often do not know what they are looking for, but they have a specific location in mind for reviewing a range of data.
  – Explore: looking for outliers is a typical case when users are not exactly sure how to start the study (Shneiderman, 1996).
- Query:
  – Identify: specific references to single individuals can be obtained from filtering.
  – Compare: comparisons among different patients can be achieved from several visualization methods.
  – Summarize: finally, a comprehensive view of all patients can be shown, and the statistics card can summarize some specific variables of the population being studied.

## Conclusions

InTool Explorer has proved to be very useful for its intended purpose, providing new insights into the raw data extracted from a variety of analyses performed in different patients and brain areas in AD. The application provides new interactive support (including other tools previously developed by the authors, e.g., Morales et al., 2011, 2012; Brito et al., 2013; Toharia et al., 2014, 2016), which improves and facilitates neuroscientists’ research.

Common tools used for analyzing raw data of the dataset presented here are basic spreadsheets and isolated visualization tools, which do not offer the same level of insight. InTool Explorer allows data to be compared quickly and easily, which was not possible with other tools. In clinical practice, a lot of information is obtained from each patient (diverse medical tests, cognitive-behavioral evaluations, etc.), which produces a huge volume of data. Applying exploratory tools, such as InTool, provides the opportunity to visualize all the information as well as manipulate it, filter variables, and explore new hypotheses. It is expected that further experiments with other datasets, similar to the ones presented in this article, will allow new hypotheses about AD and other neurological conditions to be established, thereby leading to a better understanding of these conditions.

Another significant contribution of this tool is its functional design, which makes it possible to easily combine multidimensional variables and to explore their possible relations by multiple plots. Since a large volume of data is generated, errors often appear at late stages of the research. InTool Explorer allows fast visualization of the data, detection of errors, and re-evaluation to establish new hypotheses or new lines of research. Thus, in the laboratory, this tool provides a new opportunity to study and analyze neuroscience data prior to any statistical analysis.

Finally, as mentioned above, the system was designed to facilitate the integration of new tools for visualization and analysis. According to the users’ needs, we have identified a set of statistical tests and visualizations to be implemented:

– Multi-factor ANOVA: the currently available test only considers one quantitative variable to be compared by Student’s *t*-test or U-Mann Whitney. Multi-factor ANOVA compares the means of quantitative variables analyzing their variances and allows determination of the effect of multiple factors (qualitative variables).
– Treemaps: this visualization represents data as rectangles in a hierarchical structure of the data, while displaying quantities for each category *via* area and/or color.
– Heatmaps: in combination with the correlation test (already implemented), these maps represent a fast tool for cross-examining multivariate data.
– Chord diagrams: these diagrams can be used to show relationships among categorical data.
– Force-based diagrams: allow interaction with interconnected data items in a simple way.

We plan to include these new features in the next version of the platform. Additionally, since the platform is publicly available, in order to receive feedback from users, we have implemented an e-mailbox. This option is now available under the user’s main menu as “Contact development team.” Regarding new interactions, our main efforts will focus on improving the usability of the tool.

## Data Availability

The datasets generated for this study are available on request to the corresponding author.

## Author Contributions

All authors conceived the project. DF acquired the data, contributed to the design of the tool and validations, and drafted the figures. MG designed the software tool implementation, carried out computational experiments and validations, and drafted the article. CT and ALT contributed to software tool implementation. JM designed and implemented a preliminary version of the tool. ÁR and LP supervised the design, development and implementation of the tool. JDF contributed to the design of the tool and supervised validations. LA-N contributed to the design of the tool, supervised validations and drafted the article. All authors contributed to the final manuscript.

## Conflict of Interest Statement

The authors declare that the research was conducted in the absence of any commercial or financial relationships that could be construed as a potential conflict of interest.
